# Bidispersity Improves the Toughness and Impact Resistance of Star-Polymer Thin Films

**DOI:** 10.1021/acsmacrolett.3c00671

**Published:** 2024-02-19

**Authors:** Utku Gürel, Sinan Keten, Andrea Giuntoli

**Affiliations:** † University of Groningen, Zernike Institute for Advanced Materials, Nijenborgh 4, 9747AG Groningen, The Netherlands; ‡ Department of Civil and Environmental Engineering, Northwestern University, 2145 Sheridan Road, Evanston, Illinois 60208-3109, United States; ¶ Department of Mechanical Engineering, Northwestern University, 2145 Sheridan Road, Evanston, Illinois 60208-3109, United States

## Abstract

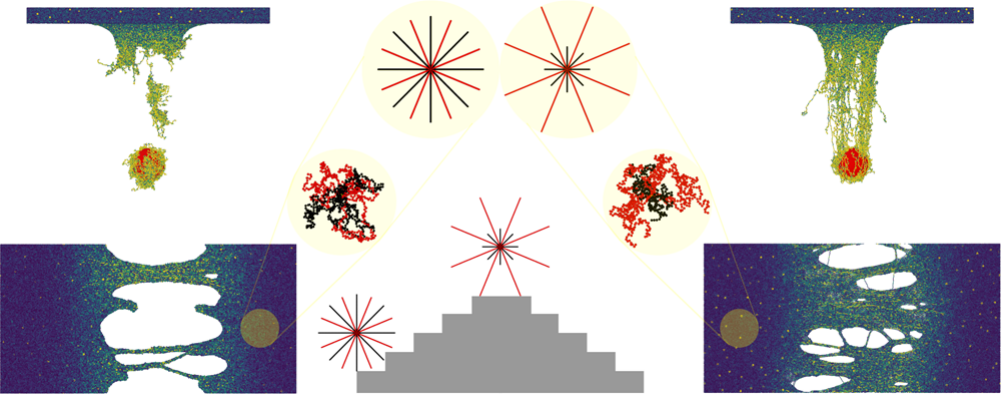

Branched polymer architectures are used to tune the mechanical properties of impact-resistant thin films through parameters, such as chain length and grafting density. While chain dispersity affects molecular properties, such as interpenetration and entanglements, structure–property relationships accounting for dispersity are challenging to obtain experimentally and are often neglected in computational models. We employ molecular dynamics simulations to model the high-rate tensile elongation and nanoballistic impact of thin films composed of bidisperse star polymers with varying arm lengths. We find that, at fixed molecular weight, high dispersity can significantly enhance the toughness and impact resistance of the films without decreasing their elastic modulus. Bidisperse stars with fewer longer arms are less entangled, but stretch and interpenetrate for longer times during crazing, leading to increased toughness. These findings highlight controlled dispersity as a design strategy to improve the mechanical properties of polymer composites across Pareto fronts.

Polymer thin films have become a focal point of interest, as they can be manufactured easily and with low cost.^1^ These materials possess favorable physical properties, such as flexibility and lightness.^2^ They have shown to be helpful in multiple fields, including protective coatings,^3^ electronics,^4^ and food packaging.^5^ Alongside other chemical aspects, the tailored mechanical attributes of polymer films are essential for their utilization in the development of impact-resistant materials.^6−8^ The use of branched polymers and polymer-grafted nanoparticles (GNPs) in thin films has recently garnered significant attention due to their unique architecture. Among these, star polymers, characterized by a central core with multiple branching arms, are distinguished by their unique topological properties. The theoretical framework addressing the architectural parameters of star polymers is well-established, especially how molecular topology influences the segmental dynamics and, consequently, the glass transition of star-polymer melts^9−11^ and thin films.^12−14^ A similar framework can be employed for the polymer relaxation and structure of GNPs composites.^15−17^ The high tunability given by nanoparticle size, polymer grafting density in the corona, and polymer chain length leads to composites with superior mechanical performance,^18−20^ particularly in comparison with traditional composites where bare nanoparticles are often not well dispersed.^21^

In laser-induced projectile impact tests (LIPIT), in particular, Chen et al. recently showed that the increase in the GNP film toughness is caused by the combined effects of entanglement density and nanoparticle jamming,^22^ which might be caused by the expansion of the interpenetrated corona during local heating.^23^ Hyon et al. also attribute the increased performance of GNPs in LIPIT experiments to adiabatic shock heating and visco-plastic film deformation.^24^ Molecular models are highly complementary to these experimental results and are able to perform LIPIT-like simulations with molecular-resolution insight into the material deformation.^25−28^ From a modeling perspective, we point out that tensile tests examining crazing and fracture in model star polymer films^29^ and in model GNP films^20^ display qualitatively the same behavior in the stress curves. From a practical standpoint, these two polymeric building blocks exhibit similar topological and mechanical properties. In our previous work, we demonstrated that the impact resistance of thin films is linked to their toughness, and this relationship can be adjusted by tuning the star topology.^29,30^ Similarly, Ethier et al. showed that the thin film toughness can be optimized by modifying the GNP topology. They attribute the increased toughness at moderate graft density to increased interdigitation and interparticle entanglement.^31^

Although computer simulations have proven useful to better understand the molecular details of polymer films under deformation, the effect of chain dispersity remains under-explored. Identifying the impact of dispersity is crucial as it affects the interactions between polymers in ways that do not manifest in monodisperse systems. Von Ferber et al. studied the effect of arm number dispersity on the effective interactions for star polymer solutions and showed that this potential is logarithmic, with a dependence on the core–core distance; however, the prefactor has an explicit dependence on the dispersity.^32^ Previously, Martin et al. showed that introducing dispersity in the graft length can be used to establish dispersions of GNPs in a polymer matrix, which would not be possible with monodisperse grafts.^33^ Bachhar et al. extended the Daoud–Cotton model^34^ to GNPs by introducing dispersity in the GNP size and grafted chain length and showed that increasing chain length dispersity leads to well-dispersed GNP solutions.^35^ In a broader context, controlling dispersity could enable us to explore the inherent characteristics of polymeric systems as an additional knob.^19^ While the cited studies explored the role of dispersity on the conformation of star polymers and GNPs, the implications for mechanical properties have yet to be investigated.

The primary goal of this work is to study the effect of dispersity on the mechanical properties of star-polymer thin films through nonequilibrium molecular dynamics simulations, performed with the LAMMPS (Large-scale Atomic/Molecular Massively Parallel Simulator) software. (https://www.lammps.org/).^36^ Polymer chain snapshots are rendered with OVITO.^37^ We use a standard bead-spring model^38^ with Lennard–Jones interactions, where the model length and time scales σ and τ can be roughly mapped to 1 nm and 10 ps, respectively.^30,39,40^ We followed closely the simulation protocol of our previous works on similar systems:^29,30^ films of thickness *h* = 20σ are equilibrated and quenched into the glassy state and are then subjected to large tensile deformations and to high-speed projectile impact, with length and time scales roughly mimicking experimental LIPIT conditions. Uniaxial deformation with a fixed transversal area is performed at a strain rate of ε̇ = 10^–3^ τ^–1^, comparable to the estimated strain rates of ∼10^8^ s^–1^ in LIPIT experiments.^41^ Simulations performed at similar strain rates have been successfully compared to experimental observations of crazing in polymer-grafted nanoparticle films.^20^ The output of the tensile test is a stress curve from which we can extract measures of elastic modulus (in the linear regime) and toughness (after plastic deformation and fracture). From the projectile impact test, we extract the curve of kinetic energy *K*(*t*) of the projectile, from which we extract the specific penetration energies *E*_p,1_^*^ = [*K*(0) – *K*(τ_1–2_)]/π*R*_p_^2^*h* and *E*_p,2_^*^ = [*K*(τ_1–2_) – *K*(∞)]/π*R*_p_^2^*h*, where τ_1–2_ is the time at which the bottom surface of the film starts deforming,^28^ and *R*_p_ is the projectile’s radius. The initial velocity of the projectile is *v⃗*_p_ = −5zσ̂/τ, roughly mapped to a velocity of 500 m/s, comparable to the experimental projectiles velocity^7,24^ in LIPIT experiments. The details and justification of our model parameters can be found in our previous papers and more extensively in the Supporting Information.

The novelty of this work is the introduction of bidispersity in the arms of the stars; see Figure 1: instead of monodisperse stars with *f* arms of length *M* attached to a core and total molecular weight *M*_w_ = *fM* + 1, we consider bidisperse chains where half of the arms are longer than the other half. We employ the following architectural code (*M*_1_, *M*_2_), where subscripts 1 and 2 refer to the shorter and longer arms, respectively. The molecular weight with the adapted convention becomes *M*_w_ = *f*(*f*_1_*M*_1_ + (1 – *f*_1_)*M*_2_) + 1, where *f*_1_ is the fraction of chains with length *M*_1_. For our case, *f* = 16 and *f*_1_ = 0.5. The stars with different dispersities have the same molecular weight of *M*_w_ = 961. We use 540 stars in each film, leading to 518940 beads in the system. We define the dispersity index (DI) as
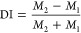
1which is a number between 0 and 1. All architectures are given in Table 1 with their dispersity indices. Here, the architecture (60, 60) is the monodisperse case, i.e., a star polymer with (*f*, *M*) = (16, 60).

**Table 1:** Star Topologies

DI	0.0	0.08	0.17	0.25	0.33	0.42	0.50	0.58	0.67	0.75	0.83	0.92
*M*_1_	60	55	50	45	40	35	30	25	20	15	10	5
*M*_2_	60	65	70	75	80	85	90	95	100	105	110	115

**Figure 1:**
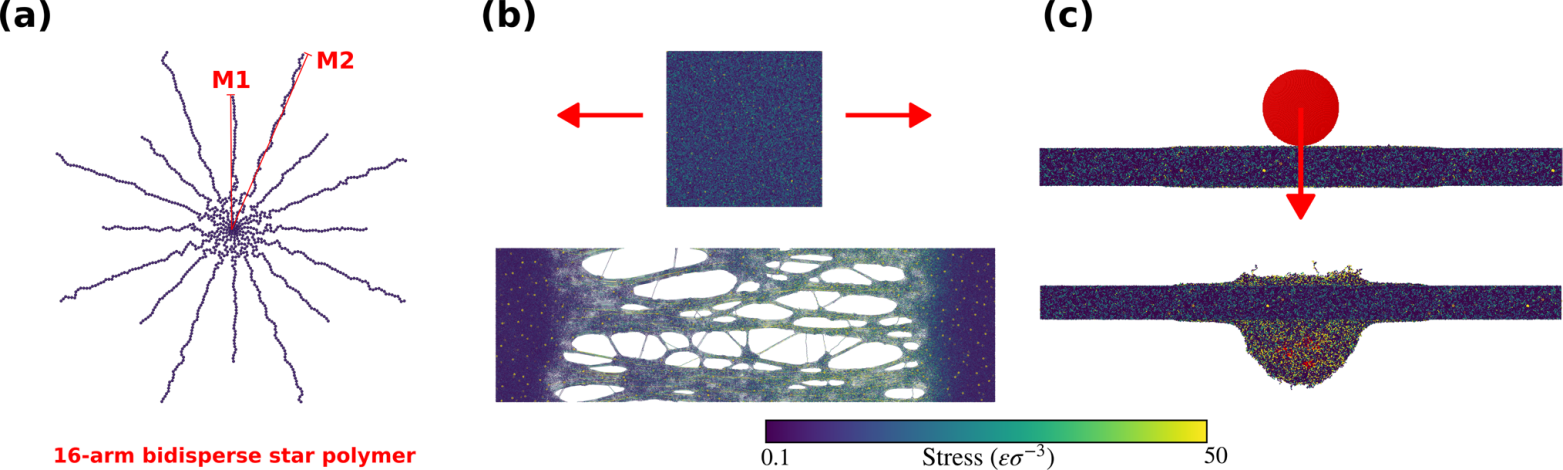
Simulation procedure: (a) Simulated bidisperse star architecture, where *M*_1_ is the shorter arm length and *M*_2_ is the longer arm length. Each star has 8 long arms and 8 short arms. We varied *M*_1_ and *M*_2_ to tune the dispersity level while keeping the molecular weight constant. The full parameter space of the architectures is given in Table 1. (b) Uniaxial deformation is applied to films to perform the tensile test from which we measure Young’s modulus and toughness. The view is from the top of the film. (c) The ballistic impact is applied to thin films to measure their impact resistance by calculating the kinetic energy loss of the projectile. The particles are colored by per-atom stress in (b) and (c).

We also compare our systems to regular star architectures with *f* = 2, 4, and 8 arms, where *f* = 2 is the linear chain and *f* = 8 corresponds to the limiting case of extreme bidispersity.

We report the stress–strain curves, Young’s modulus, and toughness in Figure 2 and compare these properties for systems with varying dispersity. We observe a local yielding point in all systems indicated by a stress overshoot. The films stretch, craze, and eventually break. The snapshots in Figure 2a show different levels of crazing for the highest DI system at the indicated strain values. Only this system exhibits strain hardening, as suggested by the second peak in the stress curve. Overall, toughness increases with increasing dispersity while modulus stays constant as shown in Figure 2b. The inset shows snapshots of polymer architectures with different dispersity indices. We can increase the toughness of thin films by a factor of ∼4.5 by only increasing the DI at fixed *M*_w_ and modulus, thus overcoming the Pareto front just via the use of bistersity in arms length. Note that a significant increase in toughness and the appearance of peaks in the stress curves is observed when the long arms of highly disperse stars go over a length of 100 beads, roughly at the beginning of the entanglement regime for the bead–spring model.

**Figure 2:**
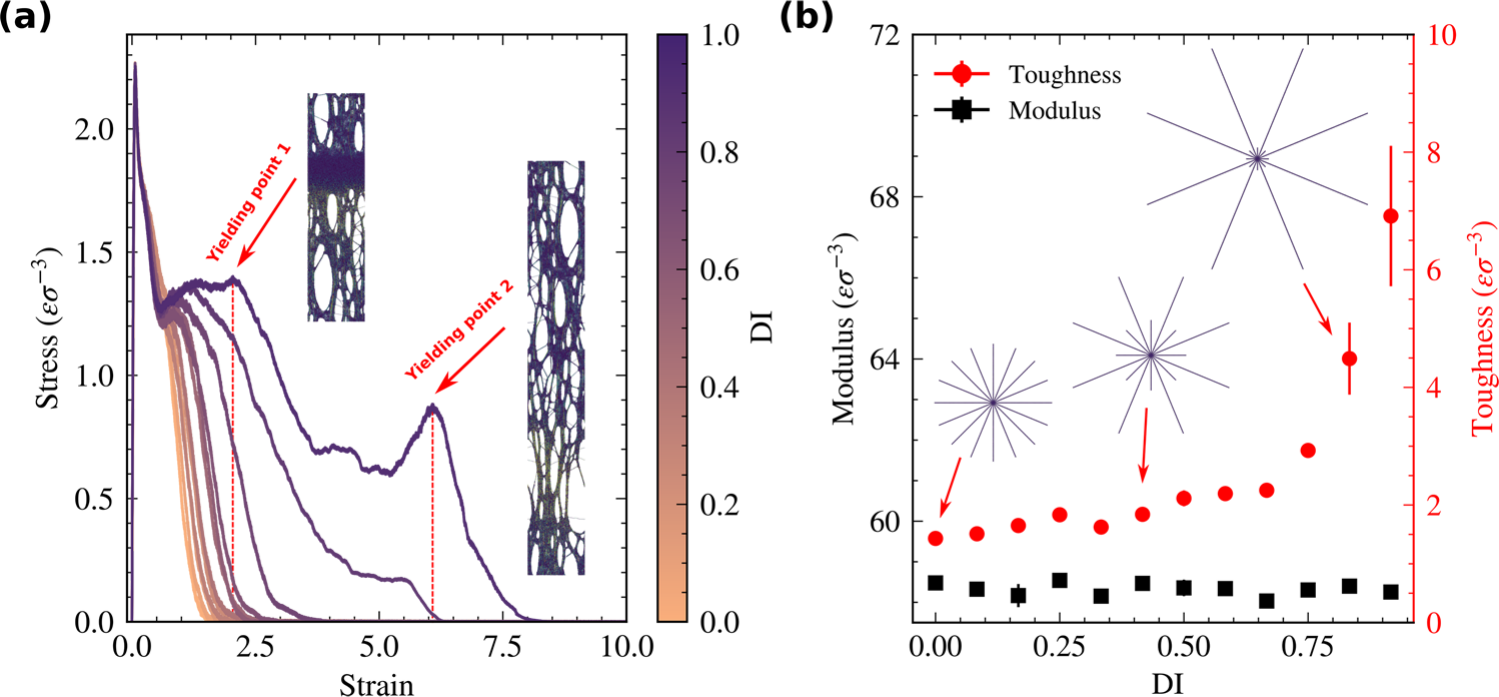
Mechanical properties of the films under deformation. Young’s modulus is measured as the slope of the stress–strain curve in the initial linear regime. Different stages of deformation are shown in the inset for the highest DI system in panel (a). Red dashed lines show the strains at which the snapshots are taken. The largest DI system comprises the longest arms in our parameter space and results in strain hardening, as suggested by the yielding points. (b) The modulus does not change as a function of dispersity for fixed *M*_w_. Toughness is measured as the integral of stress over strain. Increasing dispersity improves the film toughness by a factor of ∼4.5 while keeping the modulus constant.

At extreme dispersity, one might consider how these systems compare to, for example, monodisperse *f* = 8 stars with only long arms. We point out that the toughness of these systems can indeed be increased also by using monodisperse stars at fixed molecular weight with fewer, longer arms. However, this comes at the expense of a reduction in elastic modulus, since the grafting density of polymer chains around the core is reduced. We studied this aspect in detail at varying molecular weights in our previous work,^29^ and we report similar results for these systems in Figure S1 of our Supporting Information. The bidisperse system presented in this work allows for the tuning of toughness at fixed molecular weight without variation of the elastic modulus, thus overcoming the Pareto front.

Figure 3a shows the kinetic energy of the nano projectile over the course of the simulation for all systems. Time *t* = 0 is the time at which the velocity of the projectile starts decreasing shown by the first snapshot in the inset of Figure 3a. We compute the specific penetration energy *E*_p_^*^ into two steps, as shown to be relevant in our previous work.^28^*E*_p,1_^*^ is the energy lost in the first stage until the projectile starts deforming the bottom surface of the film (*t* = τ_1,2_) and *E*_p,2_^*^ is the energy lost during the rest of the simulation. It was previously shown that the polymer architecture affects *E*_p_^*^ in different stages of the ballistic impact.^30^ In line with the tensile tests, more disperse systems have larger values of *E*_p,2_^*^ and similar *E*_p,1_^*^ compared to less disperse films with a Spearman correlation coefficient of 0.97 between *E*_p,2_^*^ and toughness.

**Figure 3:**
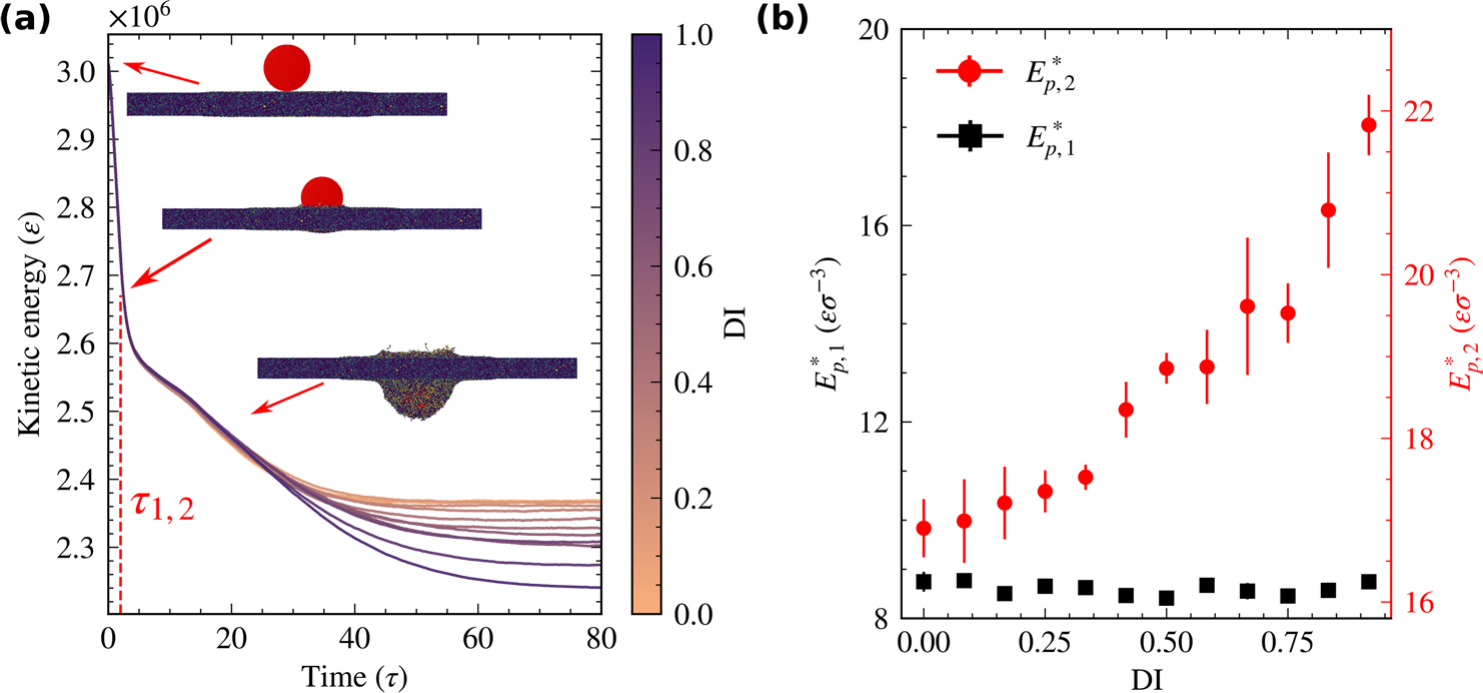
Impact resistance of thin films. (a) The kinetic energy of the projectile for different films under ballistic impact. τ_1,2_ shows the time when the bottom surface of the film starts deforming. Different stages of impact are shown in the inset for the highest DI system. (b) Normalized kinetic energy losses of the projectile under ballistic impact. *E*_p,1_^*^ marks the loss in the kinetic energy during the initial compression from *t* = 0 to *t* = τ_1,2_ = 2τ). *E*_p,2_^*^ is the energy loss during the remaining impact time. Star architecture highly affects these penetration energies at different stages.^29,30^ Increasing dispersity improves the impact resistance of thin films at the late absorption stage while keeping the early absorption energy constant. *E*_p,2_^*^ and toughness are correlated with a Spearman coefficient of 0.97.

One would intuitively expect that longer chains lead to an increase in toughness due to increased interpenetration. Still, the reduction of these long chains in our bidisperse stars could, in principle, have the opposite effect. It is then crucial to observe the arrangement and interpenetration of individual chains during deformation of the films. We calculated the orientation of individual bonds and the average number of entanglements per star arm. The bond orientation parameter is defined as
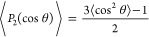
2where *P*_2_ is the second-order Legendre polynomial, θ is the angle between the bond vector and the unit vector *x̂*. The average ⟨·⟩ is taken over all bonds in the system. Figure 4a shows the evolution of *P*_2_ during the tensile test where *x̂* is the direction of the applied deformation. ⟨*P*_2_⟩ takes values between −0.5 and 1 where ⟨*P*_2_⟩ = −0.5 indicates a perpendicular alignment to the deformation direction, and ⟨*P*_2_⟩ = 1 has a perfect parallel alignment. Under equilibrium conditions, ⟨*P*_2_⟩ becomes 0, indicating a random orientation without any directional preference. Highly dispersed films reach larger values of *P*_2_ with increasing strain before they break. Note that peaks of *P*_2_ values roughly correspond to peaks in the stress curves at yielding points (Figure 2a), as shown by the red dashed lines in Figure 4a for the system with the highest dispersity.

**Figure 4:**
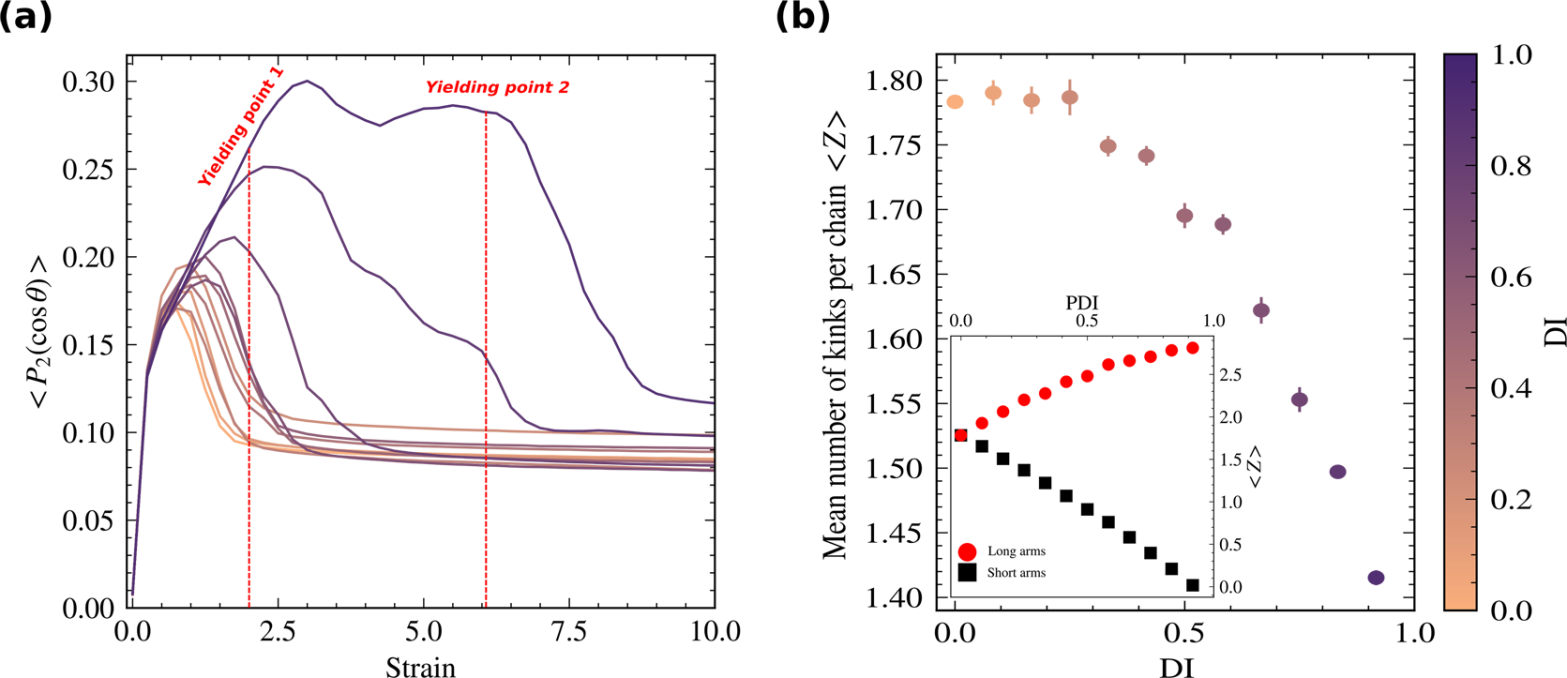
Molecular interpretation of the effect of dispersity. (a) Average bond orientation during the tensile test. We mark the values of ⟨*P*_2_⟩ at the yielding points by red dashed lines which are the same as in Figure 2. (b) Average number of kinks per chain ⟨*Z*⟩ calculated using the *Z*1+ code at equilibrium.^42^ It corresponds to the number of chain entanglements in the system. We consider the arms of the star polymers as individual linear chains for the entanglement calculation. The inset shows the mean number of entanglements per the short (black squares) and long (red circles) arms. The entanglements increase in the long arms of bidisperse stars but not as much as they decrease for the short arms, which explains the overall decreasing trend of the main panel. Despite having fewer entanglements per chain at high DI values, the strengthening of high DI thin films originates from the entanglements and stretched configuration of the fewer but longer arms (see Figure S2).

Figure 4b shows the average number of entanglements per star arm. We use *Z*1+ code to calculate the average number of kinks per chain that is used to quantify entanglements in linear chains.^42^ To be able to use this code, we remove the core of star polymers and consider the arms as individual linear chains similar to ref (31). ⟨*Z*⟩ also accounts for the self-entanglement and is measured in the equilibrium state. One might expect to have more entanglements in highly disperse systems because of the existence of long arms whose entanglement length is larger than that of linear chains of comparable sizes.^43^ However, we observe an opposite trend in Figure 4b because of the existence of short arms that are well below the entanglement length. The inset of Figure 4b indicates that the shorter arms of star polymers contribute less to the total count of entanglements for highly disperse systems. This competing effect between the less entangled short arms and more entangled long arms results in a net decreasing number of entanglements at high dispersity. On the other hand, systems with longer arms align better with the deformation direction and stay aligned longer before the film fails (see Figure S2). We attribute the enhanced mechanical properties to the longer-maintained contact between the arms during deformation.

To conclude, our study has demonstrated the potential of bidisperse star polymers in enhancing the mechanical toughness of polymer thin films to assist impact-resistant material development, as revealed by nonequilibrium molecular dynamics simulations.

We have found that star-polymer films with a higher degree of dispersity exhibit larger toughness during a uniaxial deformation, the highest degree of dispersity system having almost 4.5 times higher toughness than a monodisperse system, while keeping the elastic modulus constant. Furthermore, in our molecular analysis, we measure the bond orientation as we gradually increase the strain to conclude that longer arms retain entanglements and maintain their alignment with the deformation direction for a more extended period compared to shorter arms. The total number of chain entanglements is lower for more disperse systems, since the contribution from shorter arms is drastically reduced. These systems also have higher specific penetration energy, which correlates with their toughness.

We also highlight the similarities between our star polymers and other studies on GNPs in terms of their mechanical properties. Both systems show similar strain–stress behavior under deformation. Hence, the results we obtained for star polymers can be generalized to GNPs.

Overall, our results show that dispersity offers considerable potential for improving the design of star-polymer thin films, making a significant step forward in the search for advanced impact-resistant materials. Specifically, given the decoupling between modulus and toughness, dispersity gives a chance to break the Pareto front by independently modulating toughness for star polymer melts and also for composites based on spherical fillers with grafted polymer chains.^44^ Our findings can shed light on future research to design superior materials by manipulating dispersity as a form of defect engineering. While this study is constrained to bidisperse systems at fixed molecular weight, architectures with greater dispersity than bidisperse systems across various molecular weights can be further studied in the future.

## Data Availability

In an effort to promote Open Science practices, the LAMMPS code used to perform our simulations is available on the GitHub page of our group: https://github.com/giuntoli-group/polydisperse-star-impact.

## Supporting Information Available

Detailed simulation procedure; Toughness and Young’s modulus for comparable monodisperse systems at fixed molecular weight (Figure S1); ⟨*P*_2_⟩ parameter calculated separately for long and short chains of the bidisperse systems (Figure S2) (PDF)
