# Supplementary material for: Bidispersity Improves the Toughness and Impact Resistance of Star-Polymer Thin Films
Source: ACS Macro Lett. 2024 Feb 19;13(3):302–7. doi: 10.1021/acsmacrolett.3c00671 (PMC10956491; doi:10.1021/acsmacrolett.3c00671)
Supplement: Supplementary file 1 — mz3c00671_si_001.pdf [file mz3c00671_si_001.pdf]

# Supporting information: Bidispersity improves the toughness and impact resistance of star-polymer thin films

Utku Gürel,[†] Sinan Keten,[‡,¶] and Andrea Giuntoli[*,†]

†*University of Groningen, Zernike Institute for Advanced Materials, Nijenborgh 4, 9747AG Groningen, The Netherlands*

‡*Department of Civil and Environmental Engineering, Northwestern University, 2145 Sheridan Road, Evanston, 60208-3109, IL, United States*

¶*Department of Mechanical Engineering, Northwestern University, 2145 Sheridan Road, Evanston, 60208-3109, IL, United States*

E-mail: a.giuntoli@rug.nl

# Methods

## Model

In this work, we use coarse-grained molecular dynamics (CGMD) simulations under extreme mechanical conditions. We give an overview of the parameters studied and the simulation procedure in Fig. 1 of the main text.

We employ the bead-spring model with reduced Lennard-Jones (LJ) units where the energy ($\varepsilon$), mass ($m$), and diameter ($\sigma$) of a single bead are set to 1 leading to a unity time scale as $\tau = \sqrt{m\sigma^2/\varepsilon}$.[1] The bonded interactions in the bead-spring model are given by the harmonic potential $U_b = k(r - r_0)^2$ to represent the bonds between connected monomers, where $k = 2,500\varepsilon/\sigma^2$ is the spring constant, $r_0 = 0.9\sigma$ is the resting bond length, and $r$ is the distance between two bonded beads. The bond length of 0.9 is chosen to prevent crystallisation in the film following the same procedure as in Ref.[2] Non-bonded interactions between the beads are given by the LJ potential

$$U_{LJ}(r) = \begin{cases} \varepsilon \left[ \left(\frac{\sigma^*}{r}\right)^{12} - \left(\frac{\sigma^*}{r}\right)^{6} \right], & r \leq r_c \\ 0, & r > r_c \end{cases} \tag{1}$$

to represent the Van der Walls interactions up to a cutoff distance $r_c = 2.5\sigma$, where $\sigma^* = 2^{1/6}\sigma$ is the length scale at which the LJ potential attains its minimum value with depth $\varepsilon$. With one bead representing a Kuhn segment, the length and time units of this model can be roughly mapped to $\sigma \sim 1nm$, $\tau \sim 10ps$.[3] The bead spring model has been used by us and others in previous deformation or ballistic impact studies for star polymers[2,4] and polymer-grafted nanoparticles,[5] qualitatively capturing the effect of molecular weight or architecture on impact resistance and crazing observed in experiments.[6] CGMD simulations are carried out with the LAMMPS (Large-scale Atomic/Molecular Massively Parallel Simulator) software. (https://www.lammps.org/).[7] Polymer chain snapshots are rendered with OVITO.[8]

## Film Properties

We create thin films of star polymers with a chain dispersity. Star polymers have a core bead of unit mass and length with 16 arms attached. The standard architectural code for star polymers is given by the set $(f, M)$ where $f$ is the number of arms attached to the core, and $M$ is the number of beads on each arm. The molecular weight $(M_w)$ of the polymers with this convention is given by $M_w = fM + 1$. We consider bi-disperse chains where half of the arms are longer than the other half. We employ the following architectural code $(M_1, M_2)$ where the subscript 1 and 2 refer to the shorter and longer arms, respectively. The molecular weight with the adapted convention becomes $M_w = f(f_1 M_1 + (1 - f_1)M_2) + 1$ where $f_1$ is the fraction of chains with length $M_1$. For our case, $f = 16$ and $f_1 = 0.5$. The stars with different dispersities have the same molecular weight of $M_w = 961$. We use 540 stars in each film, leading to $518,940$ beads in the system. We define the dispersity index (DI) as

$$DI = \frac{M_2 - M_1}{M_2 + M_1}, \tag{2}$$

which is a number between 0 and 1. We also compare our systems to regular star architectures with $f = 2, 4, 8$ arms where $f = 2$ is the linear chain and $f = 8$ corresponds to the limiting case of extreme bidispersity.

## Film preparation

We randomly place the desired number of star polymers in a periodic simulation box in the $x$ and $y$ directions with fixed walls in $z$. The walls interact with the system through a $9-3$ LJ potential along the $z$-axis. The simulation box is then squeezed into a thin film of thickness $h = 20\sigma$ and lateral dimensions $L_x = L_y = 80\sigma$. This lateral size is sufficient to prevent finite-size effects during the ballistic impact[4] Based on previous work in which we varied the film thickness,[4,9] we do not expect the results presented here to change qualitatively with varying thickness, at least within ranges of thickness comparable with the characteristic size

of the polymer chains. An initial equilibration run in the liquid state is performed in the NPT ensemble (constant number of particles, pressure, and temperature) at temperature $T = 1.0\varepsilon/k_B$ and pressure $P_x = P_y = 10.0\varepsilon/\sigma^3$ for $4 \times 10^6$ steps with time-step $\Delta t = 0.005\tau$. Then we quench the system to a glassy state with $P_x = P_y = 0.0\varepsilon/\sigma^3$ and $T = 0.1\varepsilon/k_B$ well below the system $T_g \sim 0.4$,[10] where it is equilibrated for $4 \times 10^6$ steps. In the last step of the equilibration, we remove the fixed walls and equilibrate the free-standing film for $10^5$ steps. This equilibration procedure is in line with our previous studies of star-thin films[2,4] and other polymer composites.[11] The initial high temperature and pressure equilibration leads to fast mixing and annealing of the polymer chains. After removing the confining walls, the final relaxation ensures the relaxation of local stresses in the final state, particularly at the free interfaces. We run 4 independent replicas of each system, starting with different initial bead positions and velocities to minimise statistical errors. For the two systems with the largest DI, we run 8 independent replicas since the noise during the tensile test is significantly larger compared to the remaining systems.

## Tensile test

We apply uniaxial deformation to the thin films at strain rate $\dot{\varepsilon} = 10^{-3}\tau^{-1}$ along the $x$-axis while maintaining the lateral size of the simulation box in the $y$-axis constant and allowing the free surfaces in the $z$-axis to shrink. The high strain rate due to the intrinsic timescale limitations of these models is nevertheless comparable to the estimated strain rates of $\sim 10^8 s^{-1}$ in LIPIT experiments.[12] Simulations performed at similar strain rates have been successfully compared to experimental observations of crazing in polymer-grafted nanoparticle films,[5] and we previously showed that the strain-rate dependence of tensile deformations for similar composites[13] can be captured by a Cowper-Symonds model[14] and does not affect the trends observed when varying polymer architecture. Young's modulus and toughness are measured from the stress tensor components. Young's modulus is the slope of the linear fit in the initial regime, and toughness is the area under the stress curve

in Fig. 2a. The films are stretched for $2.5 \times 10^6$ steps, making sure that they all break, *i.e.* they separate into two disconnected parts after fracture. We note that this deformation protocol elongates the film while keeping a constant cross-sectional area. This has been shown[5] to be a reasonable protocol for studying the large deformation, necking, and crazing in ultrathin polymer films, where stress is more readily relaxed in the dimension of the film thickness rather than in the larger plane of the film. Since the dispersity that we introduce in our star polymers mainly affects chain entanglement and crazing, this protocol is suitable to discuss the findings of this work. The calculation of Young's modulus might be quantitatively affected by this procedure. Still, we showed before[2] that Young's modulus of star polymer thin films and bulks is inversely proportional to measures of local mobility that do not depend on the deformation protocol. For stars, this mobility and elastic response is mainly related to the grafting density and polymer packing fraction near the core. For this reason, we expect that the findings of this work relating the variations in Young's modulus when polymer dispersity is changed are not affected by the deformation procedure.

## Ballistic impact

We duplicate the film along both the $x$ and $y$ axes for the ballistic test, resulting in a surface that's four times its original size. This ensures that the film surface is sufficiently larger than the projectile, thus preventing size effects and allowing for a computationally efficient setup. We place a spherical projectile of radius $R_p = 20\sigma$ on top of the film with an initial velocity of $\vec{v}_p = -5\hat{z}\sigma/\tau$. The projectile size is chosen as in our previous study[2] to have a $R_p/h$ ratio of 1, as close as possible to LIPIT experimental studies within the possibilities of coarse-grained molecular dynamics models. $v_p$ can be roughly mapped to a velocity of $500m/s$, comparable to the experimental projectile's velocity.[6,15] Our previous work[4] showed that variations of these parameters within the same order of magnitude do not strongly affect the role of star polymer architecture on the film's properties. The projectile comprises 241260 LJ beads placed on a diamond lattice with a reduced density of 7.2 and treated as a rigid

body. We fix the boundaries of the film to prevent it from moving with the projectile. The details of this procedure are explained in Ref.[2] The interaction potential between the polymer and projectile beads is the same as non-bonded interactions. We calculate the velocity of the projectile during the impact in an NVE ensemble, conserving the total energy of the system. The projectile's kinetic energy $K(t)$ is then calculated from the velocity at each time step. We separate the change in $K(t)$ into two stages where stage 1 corresponds to the duration between $t = 0$ and the time $\tau_{1-2} = 2\tau$ at which the bottom surface of the film starts deforming. Stage 2 is the duration between $\tau_{1-2}$ and the time at which $K(t)$ stays constant. The losses in kinetic energy, normalised by the projectile surface and film thickness, are given by $E^*_{p,1} = [K(0) - K(\tau_{1-2})]/\pi R_p^2 h$ and $E^*_{p,2} = [K(\tau_{1-2}) - K(\infty)]/\pi R_p^2 h$.

## Mechanical properties of limiting monodisperse cases

It is instructive to observe the role of dispersity as opposed to simply changing the star architecture altogether in monodisperse systems. We report here the following architectures as limiting cases of extreme dispersity at a fixed $M_w$ in Fig.S1. It is possible to obtain higher toughness by reducing the number of arms in a star to have longer chains and higher entanglement; however, that compromises the elastic modulus, since the chain packing near the star core decreases, an effect that is even more pronounced in polymer-grafted nanoparticles due to the large tunability of grafting density. Our proposed strategy of tuning bidispersity allows one to achieve higher toughness without lowering the elastic response of the film.

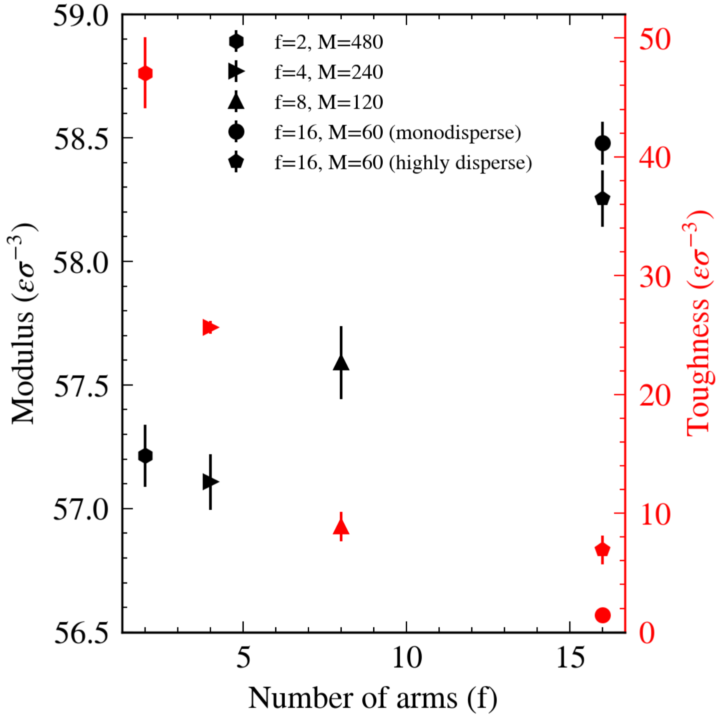


Figure S1: Modulus and toughness of limiting monodisperse cases compared to extreme bidisperse stars. The hexagonal point is a star polymer with $f = 2, M = 480$, i.e. a linear chain. The right-pointing triangle is a star with $f = 4, M = 240$, and the upper triangle is a star with $f = 8, M = 120$. The circular point is a star with $f = 16, M = 60$, i.e., the monodisperse case in our study. Finally, the pentagonal point is the most extreme bidisperse case with $M_1 = 5, M_2 = 115$.

# Orientation of short and long arms

We compute the $< P_2 >$ parameter separately for long and short arms. The contribution of long arms to the total orientation parameter is larger than that of shorter arms as seen in Fig. S2.

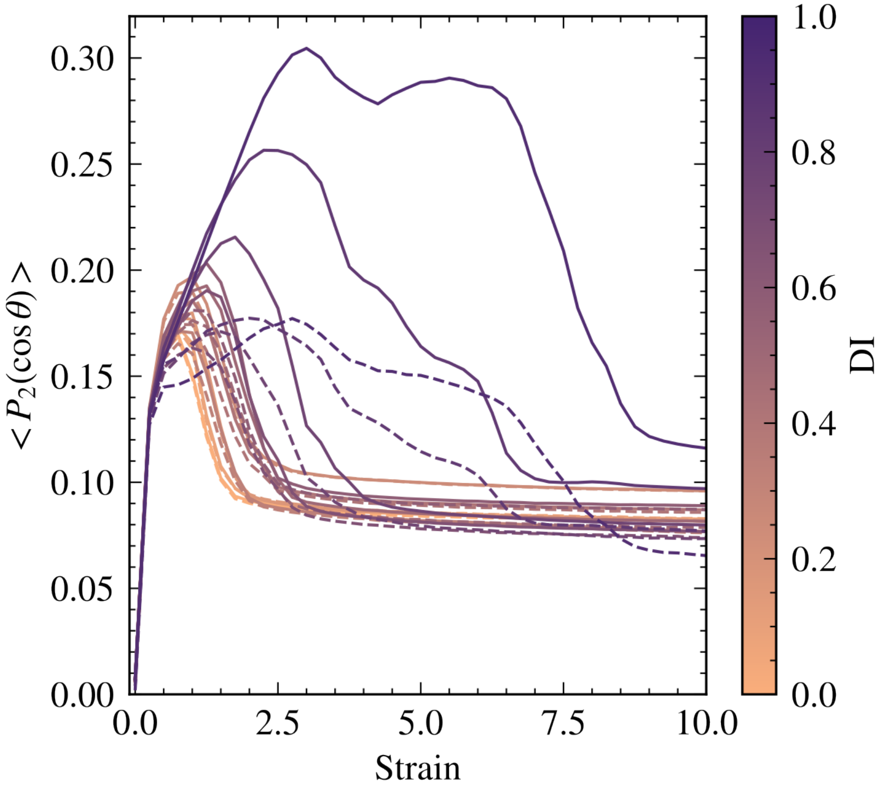


Figure S2: Average bond orientation during the tensile test for long (solid) and short (dashed) arms. Longer arms contribute more to the total orientation parameter of the arm.

# References


(1) Kremer, K.; Grest, G. S. Dynamics of entangled linear polymer melts: A molecular-dynamics simulation. *The Journal of Chemical Physics* **1990**, *92*, 5057–5086.

(2) Giuntoli, A.; Keten, S. Tuning star architecture to control mechanical properties and impact resistance of polymer thin films. *Cell Reports Physical Science* **2021**, *2*, 100596.

(3) Liu, A. Y.; Emamy, H.; Douglas, J. F.; Starr, F. W. Effects of chain length on the structure and dynamics of semidilute nanoparticle–polymer composites. *Macromolecules* **2021**, *54*, 3041–3051.

(4) Giuntoli, A.; Hansoge, N. K.; Keten, S. Star topology increases ballistic resistance in

thin polymer films. *Extreme Mechanics Letters* **2020**, *41*, 101038.

(5) Ethier, J. G.; Drummy, L. F.; Vaia, R. A.; Hall, L. M. Uniaxial deformation and crazing in glassy polymer-grafted nanoparticle ultrathin films. *ACS nano* **2019**, *13*, 12816–12829.

(6) Chan, E. P.; Xie, W.; Orski, S. V.; Lee, J.-H.; Soles, C. L. Entanglement density-dependent energy absorption of polycarbonate films via supersonic fracture. *ACS Macro Letters* **2019**, *8*, 806–811.

(7) Thompson, A. P.; Aktulga, H. M.; Berger, R.; Bolintineanu, D. S.; Brown, W. M.; Crozier, P. S.; in't Veld, P. J.; Kohlmeyer, A.; Moore, S. G.; Nguyen, T. D.; others LAMMPS-a flexible simulation tool for particle-based materials modeling at the atomic, meso, and continuum scales. *Computer Physics Communications* **2022**, *271*, 108171.

(8) Stukowski, A. Visualization and analysis of atomistic simulation data with OVITO–the Open Visualization Tool. *Modelling and simulation in materials science and engineering* **2009**, *18*, 015012.

(9) Zhu, Y.; Giuntoli, A.; Hansoge, N.; Lin, Z.; Keten, S. Scaling for the inverse thickness dependence of specific penetration energy in polymer thin film impact tests. *Journal of the Mechanics and Physics of Solids* **2022**, *161*, 104808.

(10) Zhang, W.; Douglas, J. F.; Chremos, A.; Starr, F. W. Structure and dynamics of star polymer films from coarse-grained molecular simulations. *Macromolecules* **2021**, *54*, 5344–5353.

(11) Hansoge, N. K.; Keten, S. Effect of polymer chemistry on chain conformations in Hairy nanoparticle assemblies. *ACS macro letters* **2019**, *8*, 1209–1215.

(12) Lee, J.-H.; Veysset, D.; Singer, J. P.; Retsch, M.; Saini, G.; Pezeril, T.; Nelson, K. A.;

Thomas, E. L. High strain rate deformation of layered nanocomposites. *Nature communications* **2012**, *3*, 1164.

(13) Hansoge, N. K.; Gupta, A.; White, H.; Giuntoli, A.; Keten, S. Universal relation for effective interaction between polymer-grafted nanoparticles. *Macromolecules* **2021**, *54*, 3052–3064.

(14) Cowper, G.; Symonds, P. S. Strain-hardening and strain-rate effects in the impact loading of cantilever beams. 1957.

(15) Hyon, J.; Gonzales, M.; Streit, J. K.; Fried, O.; Lawal, O.; Jiao, Y.; Drummy, L. F.; Thomas, E. L.; Vaia, R. A. Projectile impact shock-induced deformation of one-component polymer nanocomposite thin films. *ACS nano* **2021**, *15*, 2439–2446.
